# Aggressiveness predicts dominance rank in greylag geese: mirror tests and agonistic interactions

**DOI:** 10.1098/rsos.231686

**Published:** 2024-04-03

**Authors:** Sonia Kleindorfer, Mara A. Krupka, Andrew C. Katsis, Didone Frigerio, Lauren K. Common

**Affiliations:** ^1^ Konrad Lorenz Research Center for Behavior and Cognition, Core Facility of the University of Vienna, Grünau im Almtal, Vienna 4645, Austria; ^2^ Department of Behavioral and Cognitive Biology, University of Vienna, Vienna 1030, Austria; ^3^ College of Science and Engineering, Flinders University, Adelaide, South Australia 5042, Australia; ^4^ Biology Department, Kalamazoo College, Kalamazoo, MI 49006, USA

**Keywords:** anatidae, individual differences, mirror stimulation test, personality, social rank, dominance hierarchy

## Abstract

Individual differences in aggressiveness, if consistent across time and contexts, may contribute to the long-term maintenance of social hierarchies in complex animal societies. Although agonistic interactions have previously been used to calculate individuals’ positions within a dominance hierarchy, to date the repeatability of agonistic behaviour has not been tested when calculating social rank. Here, we examined the consistency and social relevance of aggressiveness as a personality trait in a free-flying population of greylag geese (*Anser anser*). For each individual, we quantified (i) aggressiveness using a standardized mirror stimulation test and (ii) dominance ranking based on the number of agonistic interactions won and lost in a feeding context. We found that individual differences in aggressiveness were significantly repeatable and that individuals’ aggressiveness predicted their dominance rank position. The flock showed a robust and intermediately steep dominance hierarchy. Social rank was higher in paired birds, males and older birds, and most agonistic interactions occurred between individuals with moderate rank differences. We suggest that selection favours aggressiveness as a personality trait associated with resource acquisition and social rank, whereby a dominance hierarchy may increase the benefits of group living and reduce costs over conflict within dyads.

## 1. Introduction

Dominance hierarchies describe the outcome of dyadic relationships between conspecifics that functionally reduce the cost of conflict over resources and thereby promote social stability [1,2]. Several components predict whether a member of a dyad will be the winner or loser of the interaction, including body size and fighting potential (e.g. tooth and claw length), and also the social experience of winning previous encounters [3]. The so-called home advantage associated with a previous winning experience can be mediated by testosterone [4,5]. Generally, dominance hierarchies are measured as the outcome of aggressive dyadic interactions and are considered to be established when subordinate individuals consistently submit to dominant individuals, although hierarchies are not always linear [6,7]. Many studies calculate dominance hierarchies per definition as the outcome of the number of agonistic interactions won versus lost, and refer to aggressiveness in relation to dominance rank [8]. However, to our knowledge, no study to date has used a cross-context validation of aggressiveness for personality [9] in relation to dominance rank.

Individuals within a population often differ consistently in their behaviour, with these variations recognized as personality differences [10]. There are typically five main personality axes considered: (i) *boldness*, an animal’s reaction to a risky situation, including predators (bold-shy); (ii) *exploration*, an animal’s reaction to a novel situation, such as a new habitat, resource or object; (iii) *activity*, an animal’s general amount of movement in a non-risky and non-novel environment; (iv) *aggressiveness*, an animal’s agonistic reaction towards conspecifics; and (v) *sociability*, an animal’s non-agonistic reaction towards conspecifics [9]. Individual differences in any of these five personality traits can influence ecologically relevant component traits (*sensu* [9]), such as dominance rank [11]. In turn, these component traits may influence important biological outcomes such as reproductive success and survival [12–15], which ultimately contribute towards an individual’s fitness.

Natural selection is expected to favour personality traits that maximize fitness [16]. As the aggressiveness axis measures agonistic interactions between conspecifics [9] and the social dominance rank predicts the uneven access to or distribution of resources among conspecifics [1], we are interested in the association between aggressiveness and dominance rank. Previous studies have measured the relationship between dominance rank and behavioural variables, such as boldness, exploration and composite sociality index [17–19]. These have generally found that more proactive (i.e. bolder, more exploratory) individuals have higher dominance rank, but that social and ecological contexts affect the strength of this association. From a mechanistic perspective, male rhesus macaques (*Macaca mulatta*) that engaged in many agonistic interactions also had higher testosterone concentration and higher dominance rank [20]. Several studies have measured glucocorticoid regulation by the hypothalamic-pituitary-adrenal axis in relation to dominance rank [21,22] and generally found higher basal cortisol in low-ranking individuals and individuals facing uncertain social contexts.

Few studies focus exclusively on the aggressiveness personality axis in relation to dominance rank, even though this personality axis measures patterns of social intolerance (e.g. agonistic interactions) between conspecifics and may be an apposite test of selection on personality associated with resource access (e.g. dominance rank)—more so than, for example, reaction norms to predators or novel situations. Of course, subsequent tests of behavioural syndromes may reveal suites of correlated traits associated with dominance rank [23]. To be considered a personality trait, a behavioural response norm such as aggressiveness needs to be measured across time and contexts. In adult superb fairywrens (*Malurus cyaneus*), response to mirror stimulation tests was consistent across time [24,25], and cross-context measures of aggressiveness have been validated in Darwin’s small ground finches (*Geospiza fuliginosa*), where response to a simulated intruder (mirror stimulation test) correlated positively with response to a simulated intruder in the territory (playback test) [26]. Given that dominance hierarchies rely on observations of winners and losers in dyadic interactions, it logically follows that more aggressive individuals should also be more dominant, if more aggressive individuals are more likely to initiate and win dyadic interactions. This is not necessarily the case, however, as aggressiveness in a standardized mirror stimulation test is expressed in response to a seemingly size-matched opponent (its mirror image). Based on condition-dependent aggression, we would expect all individuals to show similar aggressiveness in these mirror stimulation tests because they are all facing size-matched opponents [27,28]. Hence, it is not inevitable to find a relationship between standardized aggression and dominance hierarchies. Yet if these tests measure intrinsic differences in the propensity to be aggressive, then we predict that individuals responding more aggressively to size- and behaviour-matched opponents might also be more aggressive in natural dyadic encounters.

In greylag geese (*Anser anser*), males that won more agonistic interactions had higher dominance ranks (where rank 1 > rank 2, etc.), per definition [29]. However, we lack cross-context validation of aggressiveness in geese to better understand the possible role of this personality trait in predicting dominance rank position. Greylag geese are long-lived (25 years) and occur in large flocks (100+ flock members; e.g. [30]) composed of pairs, trios, homosocial partners, singletons and family units (31). Greylag geese have individually distinct faces [32] and distance calls [33], and show individual-level responses to both photos and broadcast of calls [32,33]. They can make judgements about relationships using transitive inference [34] and show the capacity for gaze following within 10 days of hatching [35]. Bystander geese increased their heart rate when observing agonistic interactions among flock mates, especially if their partners were involved [36]. Clearly, geese can recognize their flock mates and social partners. It is reasonable to assume that geese possess the behavioural and cognitive capacity to remember a dominance hierarchy and their flock mates’ relative position in the hierarchy, as found in ravens (*Corax corax*) [37,38].

In this study, we ask if aggressiveness in males and females predicts an individual’s dominance rank position in greylag geese. To quantify aggressiveness scores, we recorded agonistic interactions during mirror stimulation tests (simulated conspecific intruder; e.g. [24,39,40]) and tested whether geese exhibited consistent differences in their aggressive behaviour towards the mirror and flock mates [9]. As dominance rank functions to reduce the cost of conflict over limited resources, we examined patterns of agonistic interactions in feeding and non-feeding contexts [29,41–43]. We ask: (i) is there cross-context consistency in an individual’s aggressiveness response measured in a mirror stimulation test and the number of agonistic interactions? (ii) does the number of agonistic interactions change across feeding and non-feeding contexts, and does group size at clumped food sources predict the number of agonistic interactions? (iii) is there a robust dominance hierarchy within a flock of greylag geese? and (iv) do life-history traits (sex, age and pairing type—heterosexual, homosocial or unpaired) predict dominance rank and agonistic interactions?

## 2. Methods

### 2.1. Study site

We studied a free-flying flock of greylag geese that have been habituated to humans across 50 years at the Konrad Lorenz Research Center (47°48'49.7412" N, 13°56'51.72" E), located in the riverine valley Grünau im Almtal, Upper Austria. This flock (flock size has ranged from 92 to 144 geese) was introduced to the valley by Konrad Lorenz and colleagues and has been monitored continuously since 1973 [44,45]. Most geese (98%) are marked with an individually numbered aluminium ring and a unique combination of colour bands. The unbanded birds (2%) are distinguished by facial markings [32]. The flock is non-migratory, as they are supplemented with grain and pellets in six feeding troughs (each 1.5 m × 0.2 m) twice per day (morning and late afternoon) throughout the year. During the daily feeding, members of the flock are monitored for presence, social status, pairing status, pair partner and reproductive success [45]. Therefore, the life history and current social status of each bird within the flock are known.

### 2.2. Mirror stimulation test (aggressiveness)

In September 2021 and July 2023, we conducted mirror stimulation tests during morning feeding. Responses to mirror stimulation tests are commonly used to determine individual aggressiveness as a measure of personality that is consistent across contexts [9,25,46]. To habituate birds to the mirror stations, upright wooden boards were placed adjacent to the feeding troughs, and each had a tray (30 cm × 20 cm) of food in front of the board for 3 days. We used stones, collected from the river shore adjacent to the feeding area, to demarcate 1 and 2 m radii in front of each mirror. On the 4th and 5th days, the boards were replaced with mirrors and the food trays were filled. Geese moved freely throughout the feeding area and approached the mirrors voluntarily, so not all geese were sampled on one day, and not all geese were sampled multiple times. In addition, it was not possible to control how many geese were in the vicinity of the mirror at any one time. Each trial began when a focal goose entered within 2 m of a mirror and lasted for 5 min. During this time, we recorded the goose’s latency to approach within 1 m of the mirror and the minimum distance (m) to the mirror. An approach within 2 or 1 m of the mirror was scored when the goose stepped on the 2 or 1 m mark, respectively. We scored the first approach per individual per day. We collected mirror responses from 83 individuals (41 females, 51 males and one unknown sex), with a total of *n* = 137 responses (1–3 observations per individual, mean = 1.65 ± 0.08, one trial = 41 individuals, two trials = 30 individuals and three trials = 12 individuals). Multiple observations per individual were collected to estimate the repeatability of the behaviour across time, a key component of personality [9,47].

### 2.3. Agonistic interactions

We monitored agonistic interactions between geese during morning feeding at the six food troughs and ad hoc at other sites in July 2023. We recorded all observed agonistic interactions during feeding for 30 days, including the type of interaction (bite, chase, hiss, neck lunge and peck), interaction donor identity (ID; the bird that performed the behaviour) and interaction receiver ID. In some cases, the donor, receiver or both could not be identified; these interactions were still recorded. In addition, across the month we conducted 81 scan samples of goose behaviour, during which we noted the number of geese in the group (a group was defined based on the minimum distance of 30 m between the most distal goose and the next nearest goose) and goose behaviour (resting, moving, preening and feeding). For 10 min following each scan sample, agonistic interactions between geese were recorded ad hoc by two observers (S.K. and M.A.K.) and assigned to the preceding scan. We collected 1511 ad hoc agonistic interactions involving 111 individuals (41 females, 68 males and two unknown sex). Across 7 days, a total of 879 agonistic interactions could be allocated to a particular scan sample. There were 105 recorded donors of agonistic interactions (40 females, 65 males; 1–66 donated interactions per individual, mean = 13.44 ± 1.15).

### 2.4. Dominance hierarchy

To calculate the dominance hierarchy of the flock during July 2023, we used the randomized Elo-rating method implemented in the R package aniDOM v. 0.1.5 [48]. Traditional Elo-rating methods use the order of winner–loser sequences to infer hierarchies when these hierarchies are dynamic over time [49]. However, animal hierarchies are often temporally stable, so the function ‘elo_scores’ replicates the input dataset with a randomized order of interactions (1000 replications) to calculate mean individual ranks and 95% confidence intervals (CI) from the calculated Elo-scores [49]. Winners were defined as the individual that donated the agonistic interaction and losers were the individual that received the agonistic interaction. In our July 2023 sample, losers were not observed to challenge winners and in all cases moved away. We evaluated the steepness of the hierarchy by plotting the probability for the dominant to win in a conflict according to the difference in rank to the opponent. In steep hierarchies, this probability quickly reaches 1, whereas in flat or random hierarchies, the probability remains consistently near 0.5 [49]. We quantified the robustness (or uncertainty) of the calculated hierarchy through two methods implemented in aniDOM. The first method calculates the repeatability of individual Elo-scores across randomizations (1000 randomizations). Repeatability scores above 0.8 for this method suggest intermediate-to-high steepness and high robustness of the calculated hierarchy. The second method calculates the correlation between two hierarchies inferred from splitting the original dataset in half (1000 randomizations). Repeatability scores above 0.5 for this method suggest an intermediate-to-high steepness of the hierarchy and low uncertainty.

### 2.5. Statistical analysis

All analyses were conducted in R v. 4.1.0 [50]. All quantitative variables were scaled to standardize mean to 0 and standard deviation to 1 to facilitate the interpretation of effect sizes [51]. We present model effect sizes as estimates ± standard error (s.e.), using the *summary* function in ‘lme4’ v. 1.1.33 [52]. We report *χ* ^2^ and *p*-values from the ANOVA table of deviance, using type III *χ* ^2^ tests implemented in the package ‘car’ v. 3.0.12 [53]. We extracted predicted values using the *ggpredict* function in the package ‘ggeffects’ v. 1.2.3 [54] and plotted using ‘ggplot2’ v. 3.3.5 [55].

#### 2.5.1. Aggressiveness

We calculated aggressiveness for each bird using their response to the mirror stimulation test based on latency (s) to 1 m and minimum distance (m) to the mirror. The adjusted repeatability (R) of individual differences in aggressiveness (latency and minimum distance) was calculated using the package ‘rptR’ v. 0.9.22 [56,57], which uses linear mixed models (LMMs) implemented in ‘lme4’. Individual ID was included as the random effect and grouping variable, and the order of mirror response (1–3) was included as a fixed factor. The significance of the calculated *R* was tested using likelihood ratio tests against the null hypothesis that *R* = 0, and we calculated the 95% CI using parametric bootstrapping (1000 iterations). We calculated repeatability using all individuals irrespective of the number of mirror trials, as all individuals contribute to estimating the total population variance [58]. We used linear models (LMs) to examine the association between aggressiveness and the number of donated and received interactions.

#### 2.5.2. Agonistic interactions and group size

Using the scan sample data, we used generalized LMMs with Poisson distribution errors to assess the effect of group size on the number of agonistic interactions during feeding and non-feeding (resting, preening and moving). Models of linear, quadratic and exponential relationships of group size, the number of geese feeding, and the number of geese not feeding were explored, and the model of best fit (exponential) was selected by comparing Akaike information criteria. As multiple observations occurred within a single day, the date was included as a random effect.

#### 2.5.3. Predictors of dominance rank and number of interactions

We extracted the calculated dominance rank for each flock member to assess which traits determine rank and the number of donated or received agonistic interactions. We analysed the effects of sex, hatch year and pair type (heterosexual, homosocial and unpaired) on (i) dominance rank, using LMs; and (ii) proportion of agonistic interactions donated of the total agonistic interactions an individual was involved in, using a generalized linear model (GLM), with the *cbind* function and quasibinomial distribution to correct for overdispersion. To determine if mean aggressiveness predicted dominance rank, we used a LM with mean aggressiveness (latency and minimum distance) as the fixed factor. For the proportion of donated agonistic interactions, we included the interaction between rank and sex, rank and hatch year, and rank and pair type in the model. Non-significant interaction effects were removed from the final model using a backwards stepwise procedure.

## 3. Results

### 3.1. Agonistic interactions and mirror response

We collected 1511 ad hoc agonistic interactions involving 111 geese across 30 sampling days. Of all collected agonistic interactions, 1411 had both donor and receiver IDs recorded, and 100 only had donor ID. Interactions included 68 male and 41 female (plus two immature birds of unknown sex), 72 heterosexually paired birds, 11 homosocially paired birds and 28 unpaired birds. The mean age of the flock members was 7.4 ± 0.5 (range: 0.5–23 years). Three (male) geese known to be in the flock were not recorded as the donor or receiver of any agonistic interactions but were retained in all analyses.

**Figure 1.**
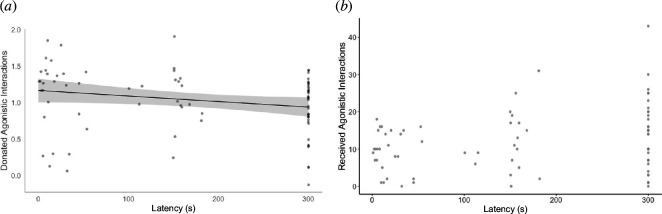
The relationship between aggressiveness (latency to approach mirror, seconds) and the number of agonistic interactions (log-transformed) that an individual (*a*) donated and (*b*) received. Raw data are presented as circles. Full model output in table 1. Shaded area in panel (*a)* represents 95% CI around the marginal effect regression line.

We collected 137 responses to the mirror simulation test from 83 individuals (41 females, 51 males and one unknown sex). The mean latency to approach within 1 m was 159 ± 12.1 s (range: 1–300), and the mean minimum distance was 81 ± 6.5 cm (range: 0–190). An individual’s response to the mirror stimulation test was significantly repeatable (latency: *R* = 0.278 ± 0.12, 95% CI: 0.031–0.511, *p* = 0.011; minimum distance: *R* = 0.279 ± 0.12, 95% CI: 0.046–0.510, *p =* 0.006). Mirror response predicted the number of agonistic interactions an individual donated (table 1): namely, individuals that approached the mirror faster also donated significantly more agonistic behaviours to conspecifics (−0.09 ± 0.05, *p* = 0.048; table 1*a*; figure 1*a*). Conversely, the minimum distance to the mirror did not predict the number of donated agonistic behaviours (table 1*a*). Individuals that reacted more aggressively towards the mirror did not receive fewer agonistic interactions compared to less aggressive individuals (latency: −0.06 ± 0.04, *p* = 0.152; minimum distance: −0.07 ± 0.04, *p* = 0.076; table 1*b*; figure 1*b*).

**Table 1.** Output from linear regressions testing the association between two measures of aggressiveness during the mirror stimulation test ((i) latency to approach within 1 m of mirror and (ii) minimum distance to mirror) and (*a*) the number of agonistic interactions donated (log-transformed), and (*b*) the number of agonistic interactions received (log-transformed). (Bold values indicate statistical significance (*p* < 0.05).)

	estimate	s.e.	*t*	*p*
(*a*) no. donated				
(i) latency				
intercept	1.031	0.05	21.99	**<0.001**
latency	−0.095	0.05	−2.00	**0.048**
(ii) minimum distance				
intercept	1.031	0.05	21.62	**<0.001**
minimum distance	−0.052	0.05	−1.09	0.281
(*b*) no. received				
(i) latency				
intercept	0.997	0.04	24.90	**<0.001**
latency	0.058	0.04	1.45	0.152
(ii) minimum distance				
intercept	0.997	0.04	25.07	**<0.001**
minimum distance	0.072	0.04	1.80	0.076

### 3.2. Behavioural context of agonistic interactions

**Figure 2.**
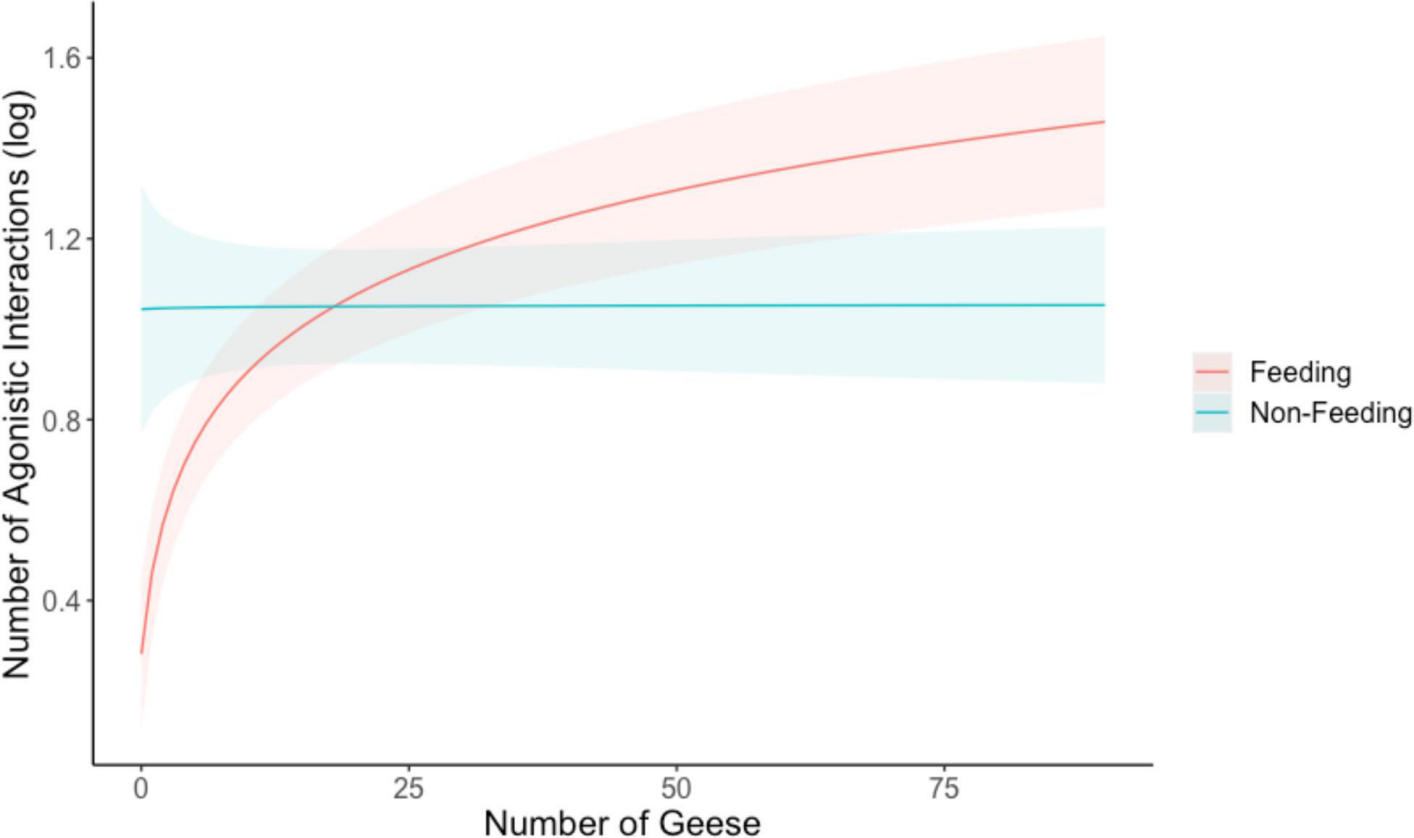
Predicted values of the effect of the number of geese on the number of agonistic interactions (log-transformed). Feeding and non-feeding contexts are shown separately. Full model output in table 1. Shaded area represents 95% CI around the marginal effect regression line.

We collected 81 scan samples across 7 days and analysed 879 agonistic interactions in relation to group size and the number of feeding versus non-feeding geese. Total group size during feeding ranged between 10 and 91 individuals (48.8 ± 3), with an average of 17.1 ± 3 feeding (0–90) and 31.7 ± 2 non-feeding (resting, preening and moving combined, 0–90). We found significant quadratic relationships between the number of agonistic interactions and both total group size (0.64 ± 0.05, 95% CI: 0.534–0.740, *p* < 0.001; table 2*a*) and the number of geese feeding (0.80 ± 0.06, 95% CI: 0.691–0.912, *p* < 0.001; table 2*b*). The number of agonistic interactions increased rapidly until a group size of approximately 40 individuals and the number of feeding geese of approximately 25, after which the rate of increase declined (figure 2). There was no association between the number of non-feeding geese and the number of agonistic interactions (−0.032 ± 0.03, 95% CI: −0.093 to 0.031, *p* = 0.318; table 2*b*; figure 2).

**Table 2.** Exponential regression using generalized linear mixed models (with Poisson error distribution) to test the effects of (*a*) group size, and (*b*) number of geese feeding and the number of geese not feeding (resting, preening and walking) on the number of agonistic interactions. (Date was included as a random effect. Bold values indicate statistical significance (*p* < 0.05). *Notes.* Variance for random effect date for (*a*) 0.478 ± 0.69 and (*b*) 0.159 ± 0.40).

	estimate	s.e.	*z*	*p*
(*a*) group size				
intercept	2.011	0.24	4.052	**<0.001**
log(group size)	0.635	0.05	12.088	**<0.001**

	estimate	s.e.	*t*	*p*
(*b*) no. feeding geese				
intercept	1.919	0.15	12.763	**<0.001**
log(no. feeding)	0.800	0.06	14.261	**<0.001**
log(no. non-feeding)	−0.032	0.03	−0.998	0.318

### 3.3. Dominance hierarchy

**Figure 3.**
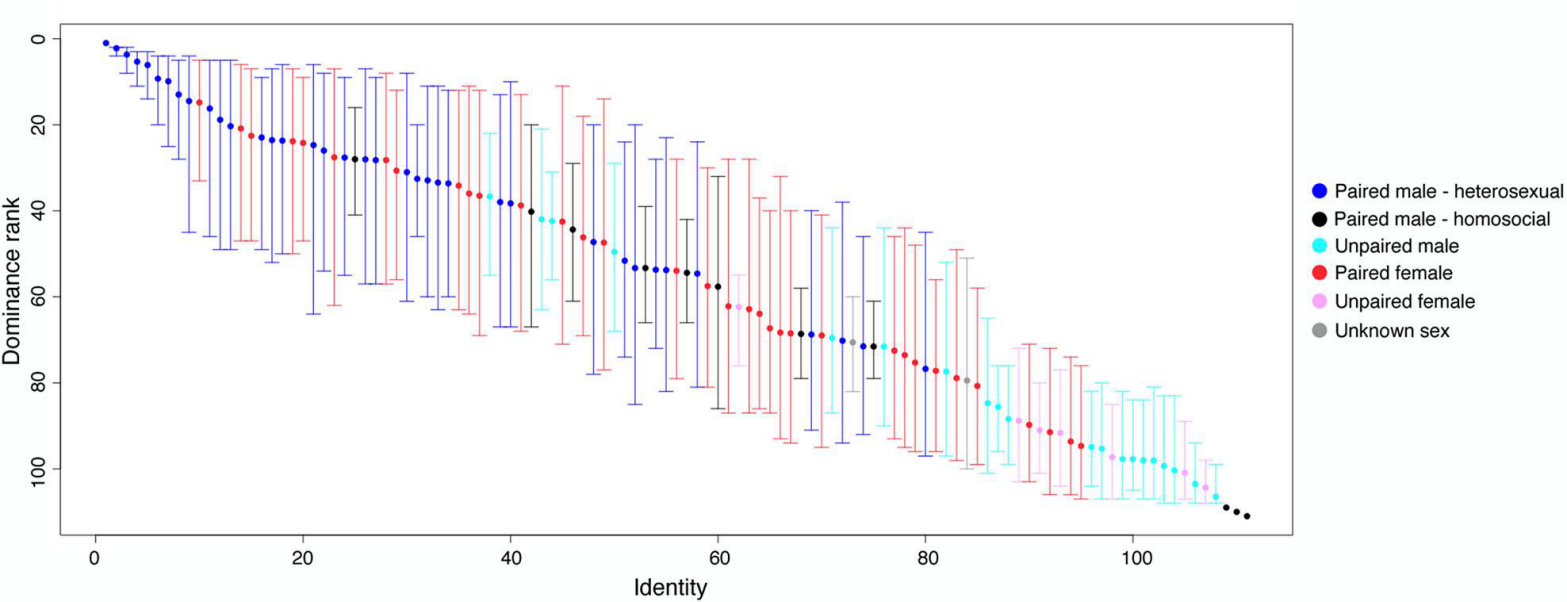
Individual dominance rank with 95% CI (1000 randomizations) for each goose in July 2023, calculated from 1411 interactions. Dominance ranks are ordered from top (upper left) to bottom (lower right). Heterosexual paired males = deep blue, homosocial paired males = black, unpaired males = cyan, paired females = red, unpaired females = pink and birds of unknown sex = grey. Three geese (Eastwood, Joe and Rosenrot, shown at bottom right) were not represented in any interactions.

**Figure 4.**
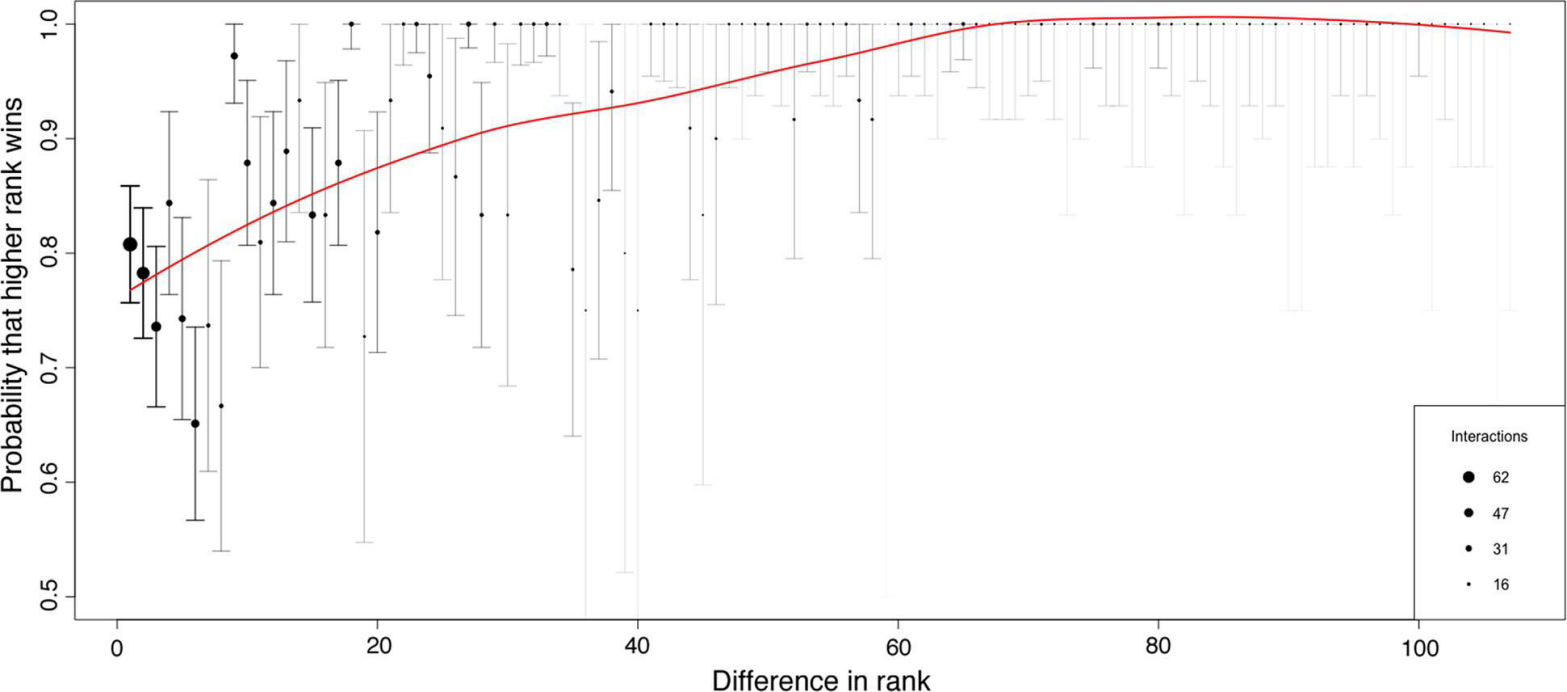
Shape of the dominance hierarchy of greylag geese in July 2023, plotting the probability for a dominant goose to win a conflict with respect to the rank difference with its opponent, with 95% CI (1000 randomizations). Point size represents the number of interactions in the dataset for each rank difference.

We found a steep dominance hierarchy between the 111 geese within the flock (figures 3 and 4). The probability for the dominant goose to win an interaction quickly increased to above 0.9 at a rank difference of 30 (figure 4). At a rank difference of approximately 60, the probability of a dominant winning the interaction was 1 (figure 4). Even at a rank difference between 1 and 10, the probability of a dominant winning the interaction was 0.75. The repeatability of the hierarchy was high: 0.89 (calculated by randomization) and 0.71 (95% CI: 0.64–0.78, calculated by splitting). These results indicate a robust, intermediate-to-high steepness of hierarchy with low uncertainty. Most interactions occurred between individuals with a rank difference of 20 or less.

### 3.4. Predictors of dominance rank and proportion of donated interactions

**Figure 5.**
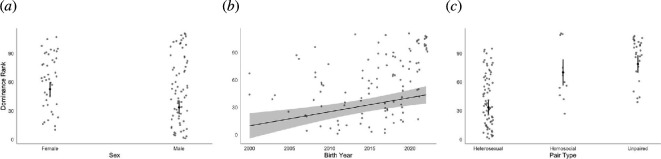
The relationship between goose dominance rank and (*a*) sex, (*b*) hatch year and (*c*) pair type. Raw data are presented as circles. Lower numbers indicate higher rank within the dominance hierarchy. Full model output in table 2*a*. Error bars in (*a*) and (*c*) represent 95% CI around the estimated marginal means, and the shaded area in (*b*) represents 95% CI around the marginal effect regression line.

We found that sex, hatch year and pairing type all predicted dominance rank (table 3*a*). Males had a higher mean rank than females (−18.98 ± 4.84, 95% CI: −28.581 to –9.377, *p* < 0.001; table 3*a*; figure 5*a*) and older birds had a higher rank than younger birds (8.26 ± 2.25, 95% CI: 3.796–12.730, *p* < 0.001; table 3*a*; figure 5*b*). Birds in heterosexual pairs had a significantly higher rank compared to homosocial and unpaired birds (heterosexual–homosocial: 35.6 ± 7.79, *p* < 0.001; heterosexual–unpaired: −45.4 ± 5.39, *p* < 0.001; electronic supplementary material, table S1; figure 5), who did not differ significantly from each other (−8.9 ± 8.34, *p* = 0.537; electronic supplementary material, table S1; figure 5).

**Table 3.** (*a*) Output from linear model testing the effects of sex (female and male), age (hatch year) and pair type (heterosexual, homosocial and unpaired) on dominance rank; and (*b*) output from generalized linear model (quasibinomial distribution^a^) testing the effects of sex, age and pair type on the proportion of agonistic interactions that were donated rather than received. (Female is the reference category for sex and heterosexual is the reference category for pair type. Sample size for all models was 109 individuals. Bold values indicate statistical significance (*p* < 0.05).)

	estimate	s.e.	*t*	sum squared	*F*	*p*
(*a*) dominance rank						
intercept	52.611	3.70	14.22			
sex—male	−18.979	4.84	−3.92	8096	15.36	**<0.001**
hatch year	8.263	2.25	3.67	7092	13.46	**<0.001**
pair type—homosocial	36.470	7.79	4.68	41 388	39.27	**<0.001**
pair type—unpaired	45.372	5.39	8.43			

	estimate	s.e.	*t*	chi squared	*p*
(*b*) donated agonistic interactions					
intercept	−0.072	0.09	−0.782		
sex—male	−0.008	0.12	−0.061	0.004	0.951
rank	−0.753	0.08	−9.703	145.63	**<0.001**
hatch year	−0.055	0.07	0.791	0.002	0.963
pair type—homosocial	0.615	0.29	2.124	15.31	**<0.001**
pair type—unpaired	0.522	0.27	1.906		
rank × hatch year	0.173	0.07	2.441	6.15	**0.013**
rank × pair type—homosocial	−1.329	0.56	−2.372	36.93	**<0.001**
rank × pair type—unpaired	−1.286	0.27	−5.019		

^a^ Dispersion parameter of quasibinomial distribution taken to be 1.506.

There was no significant effect of sex on the proportion of donated agonistic interactions (−0.01 ± 0.12, 95% CI: −0.250 to 0.234, *p* = 0.951; table 3*b*). The proportion of agonistic interactions an individual donated decreased across ranks; that is, higher-ranked geese donated more than they received and lower-ranked geese received more than they donated (−0.75 ± 0.08, 95% CI: −0.906 to –0.602, *p* < 0.001). There was a significant interaction between hatch year and rank when predicting the proportion of donated agonistic interactions (*χ* ^2^ = 6.15, *p* = 0.013, table 3*b*; electronic supplementary material, figure S1a), whereby older birds of higher rank donated proportionally fewer agonistic interactions than younger birds of higher rank (electronic supplementary material, figure S1a). However, at lower ranks, older birds donated proportionally more agonistic interactions than younger birds (electronic supplementary material, figure S1a). There was also a significant interaction between pair type and rank when predicting the proportion of donated agonistic interactions (*χ* ^2^ = 36.93, *p* < 0.001, table 3*b*; electronic supplementary material, figure S1b). At higher ranks, birds in heterosexual pairings had a lower proportion of donated agonistic interactions than homosocial or unpaired birds; at lower ranks, unpaired individuals had a lower proportion of donated agonistic interactions than heterosexual individuals, but homosocial birds did not differ significantly from the other pair types (electronic supplementary material, figure S1b).

As expected, because we used agonistic interactions to determine dominance rank, an individual’s rank also predicted its response to the mirror stimulation test. Birds of higher rank had higher mean aggressiveness during the mirror stimulation test compared to low-ranking birds: that is, they approached more quickly (8.00 ± 3.45, 95% CI: 1.138–14.853, *p* = 0.023) and more closely (7.76 ± 3.45, 95% CI: 0.890–14.631, *p* = 0.027) to the mirror (table 4; electronic supplementary material, figure S2).

**Table 4.** Output from linear models testing the association between dominance rank and two measures of aggressiveness ((*a*) latency to approach within 1 m of mirror and (*b*) minimum distance to mirror) in greylag geese (*n* = 83). (Bold values indicate statistical significance (*p* < 0.05).)

	estimate	s.e.	*t*	*p*
(*a*) latency				
intercept	51.229	3.42	14.95	**<0.001**
latency	7.996	3.45	2.32	**0.023**
(*b*) minimum distance				
intercept	51.229	3.43	14.93	**<0.001**
minimum distance	7.761	3.45	2.25	**0.027**

## 4. Discussion

In greylag geese, aggressiveness is a repeatable personality trait across both time and contexts that is associated with the number of agonistic interactions and an individual’s dominance rank position. There was a robust and intermediately steep dominance hierarchy within the flock. Males, older birds, and birds within heterosexual pairings had higher positions within the dominance hierarchy. Lower-ranking birds donated fewer and generally received more agonistic interactions, except for low-ranking younger birds and low-ranking heterosexually and homosocially paired individuals, who received fewer agonistic interactions. Significantly more agonistic interactions occurred when geese were feeding, and the number of interactions plateaued when the number of individuals feeding was approximately 25. Most agonistic interactions occurred between individuals with a rank difference of 20 or less; this suggests that geese are unlikely to initiate costly agonistic interactions with flock mates that significantly outrank them, presumably thereby keeping the number of interactions stable even when group size increases. Therefore, a dominance hierarchy, predicted by aggressiveness, may mediate the costs of living within large groups during resource acquisition, keeping costs low (fewer agonistic interactions) but benefits high (increased protection from predators). This study was conducted in the wild, using geese that were free to approach the mirror and food trays and to engage in agonistic interactions at will. Therefore, we acknowledge that the social context of these behavioural observations could have been affected by the number and identity of other flock mates in the testing area [59].

Aggressiveness during the mirror stimulation tests in 2021 and 2023 correlated with the number of agonistic interactions and dominance rank in 2023, suggesting a mechanistic pathway linking personality to dominance hierarchy [60]. These findings underscore the potential for selection to favour personality traits associated with resource acquisition [61], in this case, food resources. While some studies have measured consistency in physiological or hormonal response to social stressors and dominance rank [18,62,63], we are not aware of studies that have measured aggressiveness across time or contexts in relation to dominance rank.

In the goose flock, most conflicts occurred between flock mates of similar rank (i.e. rank differences of 20 or less). This pattern aligns with predictions for assessment strategy and the evolution of fighting behaviour [64]. The more closely ranked two individuals are, the more uncertain the winning outcome will be, and hence the greater the chance to increase rank position following the interaction. Yet the costs of the interaction would be greatest when ranks are most similar, as the fighting potential of both rivals would presumably also be similar. Aggressive males that use behavioural displays indicative of their motivational state to fight (e.g. neck lunges that indicate body size) can be assessed by the opponent. Many animals use signals to mediate aggressive interactions and reduce the costs of conflict via signalling [65,66]. Morton [67] proposed the motivational–structural rule, whereby vocalizations used in agonistic interactions should be low frequency and harsh-sounding, as opposed to vocalizations used in friendly contexts, which are predicted to be higher frequency and pure tones. Greylag geese produce soft contact calls during affiliative communication between family members and pair bonds, and produce hiss vocalizations during the conflict—but both call types need to be formally described [44]. Future research should examine component traits of the agonistic behavioural repertoire in geese, to test whether, for example, neck lunges and/or hiss vocalizations are an honest indicator of body size or motivational state in greylag geese.

The dyadic patterns of aggressiveness between individuals result in the emergent property of a dominance hierarchy [68]. Establishing a dominance hierarchy should reduce the mean number of agonistic interactions per group member, thereby maximizing the benefits of group living and reducing its costs. Because an individual’s position in a dominance hierarchy predicts its resource acquisition, social rank may influence biological fitness [61,69–72]. Notably, our non-migratory study flock is relatively small (< 150 flock members) and stable in terms of flock membership, which may help facilitate the formation of dominance hierarchies. In this study, agonistic behaviour and dominance rank were higher in paired birds (both sexes), single males and older birds, and lower in homosocial males. Perhaps aggressive males or females are preferred as partners. Being in a pair with at least one aggressive goose can increase chances of accessing resources. Conversely, paired individuals may behave aggressively as a form of ‘mate guarding’, which could be interrogated by comparing the aggressiveness of paired individuals in the presence and absence of their partner [73,74]. The finding of lower aggressiveness in homosocial male pairs could occur as the result of a skewed operational sex ratio and female preference for aggressive males. Such a pattern has been observed in blue tits (*Cyanistes caeruleus*), where unbalanced sex ratios and female aggression restricted mate choice [75]. However, a complementary explanation is that homosociality may be adaptive, given that male birds in homosocial pairs received less aggressive behaviour from other flock members [76]. Given our finding that older birds were more aggressive than younger birds, aggressiveness may be linked with longevity, although this would need to be confirmed via a longitudinal study. If there is a longevity association with aggressiveness, this could be explained by improved access to resources, which should confer a fitness benefit, as long as the cost of agonistic interactions to maintain a high dominance rank position does not escalate [77–80].

Viewed within a Tinbergian framework, future research could explore the ontogeny of aggressive behaviour in geese, which is currently not known [81]. When do goslings first express agonistic behaviour towards conspecifics [82,83], and what social or ecological conditions predict the magnitude of its expression [84,85]? In previous studies of the greylag goose, individuals with more agonistic behaviour had greater levels of circulating testosterone [86,87], which was also tested experimentally. Future research could examine how winner–loser effects mediate fluctuation in hormones and dominance rank position in this system [5,88,89], and the causal pathways for hormone concentration and aggressive behaviour in relation to sex [90]. From a functional perspective, aggressiveness has been shown to mediate access to food [91], mates [92–95] and safe nest sites [96]. From a phylogenetic perspective, the evolution of aggression has probably been shaped by both its benefits (e.g. access to resources) and its costs (e.g. risk of injury) [97]. Over evolutionary history, traits have evolved that signal aggressive intent, presumably to maximize the benefits of aggression and reduce the costs of interactions, as opponents can assess the fighting potential of rivals [98–100]. In geese, exaggerated wing displays, extended neck displays, and hiss vocalizations signal the potential for aggressive escalation and presumably evolved to mitigate the costs of aggressive encounters in this group-living species.

Here, we provide a cross-context validation of aggressiveness as a personality trait in greylag geese and find an association between aggressiveness, the number of agonistic interactions and dominance rank position. In contrast to other studies that have focused on social, cognitive or behavioural pathways underpinning dominance hierarchies, our study shows that an individual-level personality trait in a basal avian lineage is tightly associated with social rank. Our results suggest that standardized aggression can be used as a proxy for dominance. To reliably estimate a linear dominance hierarchy, researchers need to collect a 10–20 minimum ratio of observed interactions to individuals [49], which equates to 1000–2000 interactions for a population of 100 individuals. In this study, we recorded 1511 dyadic interactions for 111 greylag geese, yielding a minimum ratio of 13.6, which should reliably estimate the dominance linear hierarchy. Therefore, our findings that aggressiveness measured during a mirror stimulation test also predicted an individual’s dominance rank position support the idea that aggressiveness *per se* can potentially be used to infer social dominance at food resources. Given the difficulty of collecting sufficiently large numbers of interactions from free-ranging wild animals, our findings may be useful when estimating linear dominance hierarchies in other systems, where it may be easier to measure individual-level intrinsic aggressiveness rather than collate a large number of dyadic interactions.

## Data Availability

Data are available as electronic supplementary material [101].
